# Suicide Rates by Major Occupational Group — 17 States, 2012 and 2015

**DOI:** 10.15585/mmwr.mm6745a1

**Published:** 2018-11-16

**Authors:** Cora Peterson, Deborah M. Stone, Suzanne M. Marsh, Pamela K. Schumacher, Hope M. Tiesman, Wendy LiKamWa McIntosh, Colby N. Lokey, Aimée-Rika T. Trudeau, Brad Bartholow, Feijun Luo

**Affiliations:** ^1^Division of Analysis, Research, and Practice Integration, National Center for Injury Prevention and Control, CDC; ^2^Division of Violence Prevention, National Center for Injury Prevention and Control, CDC; ^3^Division of Safety Research, National Institute for Occupational Safety and Health, CDC; ^4^Division of Surveillance, Hazard Evaluations, and Field Studies, National Institute for Occupational Safety and Health, CDC.

During 2000–2016, the suicide rate among the U.S. working age population (persons aged 16–64 years) increased 34%, from 12.9 per 100,000 population to 17.3 (https://www.cdc.gov/injury/wisqars). To better understand suicide among different occupational groups and inform suicide prevention efforts, CDC analyzed suicide deaths by Standard Occupational Classification (SOC) major groups for decedents aged 16–64 years from the 17 states participating in both the 2012 and 2015 National Violent Death Reporting System (NVDRS) (https://www.cdc.gov/violenceprevention/nvdrs). The occupational group with the highest male suicide rate in 2012 and 2015 was Construction and Extraction (43.6 and 53.2 per 100,000 civilian noninstitutionalized working persons, respectively), whereas the group with the highest female suicide rate was Arts, Design, Entertainment, Sports, and Media (11.7 [2012] and 15.6 [2015]). The largest suicide rate increase among males from 2012 to 2015 (47%) occurred in the Arts, Design, Entertainment, Sports, and Media occupational group (26.9 to 39.7) and among females, in the Food Preparation and Serving Related group, from 6.1 to 9.4 (54%). CDC’s technical package of strategies to prevent suicide is a resource for communities, including workplace settings (*1*).

NVDRS combines data on all violent deaths (defined as those resulting from the intentional use of physical force or power, threatened or actual, against oneself, another person, or a group or community), including suicide, based on death certificates, coroner/medical examiner reports, and law enforcement reports. Data on usual lifetime occupation among 22,053 suicide decedents aged 16–64 years from the 17 states* that participated in NVDRS in 2012 and 2015 were analyzed. CDC’s National Institute for Occupational Safety and Health Industry and Occupation Computerized Coding System (NIOCCS 3.0) (https://wwwn.cdc.gov/nioccs3) was used to assign 2010 U.S. Census civilian occupation and industry codes to NVDRS decedent records based on decedents’ usual lifetime occupation and industry as reported on the death certificate. Results are reported by 2010 SOC major groups, converted from U.S. Census codes by NIOCCS. Records that could not be coded by NIOCCS were manually coded using the NIOCCS computer-assisted feature. All coding assignments were reviewed by industry and occupation coding experts for accuracy.

Suicide counts are presented by year, sex, and usual lifetime occupational group. Suicide rates were calculated using annual civilian noninstitutionalized working population counts by occupational group (based on longest job held during the previous calendar year) from the Current Population Survey Annual Social and Economic Supplement (*2*) as the denominator; 95% confidence intervals (CIs) were calculated using the National Center for Health Statistics method for death rates (*3*). The rate change from 2012 to 2015 is presented for each occupational group by sex, as is each group’s rank for rate change (i.e., where rank position 1 signifies the greatest suicide rate increase). Decedents whose NVDRS data from coroner/medical examiner reports or law enforcement reports indicated that the decedent was not employed at the time of death (unemployed, disabled, incarcerated, homemaker, or student) were excluded from rate calculations, as were decedents with military or unpaid occupations, and those with insufficient information to classify occupation. Separate analyses of suicide deaths among males in agriculture-related SOC detailed groups were conducted; such rates were not calculated for female decedents because of small numbers.

NIOCCS classified 83% (8,858 in 2012 and 9,508 in 2015) of decedent records (Table 1); this count includes those that the NIOCCS program determined to have insufficient information to classify occupation. After expert review of NIOCCS automated code assignments, 231 (3%) of 2012 records and 290 (3%) of 2015 records were recoded. The remaining 1,799 (2012) and 1,888 (2015) (17% for both years) records were coded using the NIOCCS computer-assisted feature. For 2012 and 2015 combined, 5,089 (23%) decedents were not included in suicide rate calculations because they were in the military, had unpaid occupations (e.g., did not work, homemaker, or student), or had insufficient information to classify lifetime occupation. Another 2,236 (10%) were excluded because they were not employed at the time of death.

**TABLE 1:** Procedure for Identification of analysis cohort of suicide decedents, by occupation — National Violent Death Reporting System (NVDRS) — 17 U.S. states* 2012 and 2015

Analytic procedure	2012 no.	2015 no.
Suicide decedents obtained from NVDRS data set^†^	12,811	13,967
**Excluded before assignment of occupation code**		
Aged <16 years or >64 years or missing sex	2,154	2,571
**Assignment of occupation code**		
Assigned based on decedent usual lifetime occupation	10,657	11,396
Autocoded by NIOCCS	8,858	9,508
Manually reassigned using the NIOCCS computer-assisted feature	231	290
Manually assigned using the NIOCCS computer-assisted feature	1,799	1,888
**Rate analysis**		
Decedents presumed to be in the labor force at time of death^§^	6,881	7,847

**Abbreviation:** NIOCCS = National Institute for Occupational Safety and Health Industry and Occupation Computerized Coding System.

*Alaska, Colorado, Georgia, Kentucky, Maryland, Massachusetts, New Jersey, New Mexico, North Carolina, Ohio, Oklahoma, Oregon, Rhode Island, South Carolina, Utah, Virginia, and Wisconsin.

^†^ The total number of deaths (including suicides) reported in the NVDRS data set (June 2018) for the 17 U.S. states analyzed included 19,885 decedents in 2012 and 21,884 decedents in 2015.

^§^ Decedents were not included in suicide rate calculations if they were in the military, had unpaid occupations (e.g., did not work, homemaker, or student), or had insufficient information to classify lifetime occupation. In addition, suicide decedents whose NVDRS data from coroner/medical examiner or law enforcement reports indicated no employment (e.g., retired, unemployed, disabled, incarcerated, home maker, or student) at the time of death were also excluded from rate analysis. Decedents were excluded if NVDRS current occupation information contained any of the following: “student, unemp*, not empl*, laid off, retir*, disab*, incarcer*, inmate, prisoner, homemaker, home maker, housewife, house wife, never worked, or not working.” Manual review of records excluded based on these criteria resulted in five records being reinstated (e.g., “student teacher” and two occupations listed for a decedent with just one occupation explicitly identified as retired).

In both 2012 and 2015, the largest percentage of male suicides (19%–20% of decedents) occurred among those in the Construction and Extraction group (SOC 47) (Table 2); the largest percentage of female suicides in both years occurred among decedents with unpaid occupations (29%). The largest percentage of female suicides among classifiable occupations occurred in the Office and Administrative Support group (SOC 43) in both years (15%). In both years, the highest suicide rates among males were in the Construction and Extraction group (43.6 in 2012 and 53.2 in 2015 per 100,000 civilian noninstitutionalized working persons) (Table 3). Among females, the highest suicide rates in both years were in the Arts, Design, Entertainment, Sports, and Media group (SOC 27) (11.7 in 2012 and 15.6 in 2015). Among males, the largest suicide rate increase from 2012 to 2015 (47%) occurred in the Arts, Design, Entertainment, Sports, and Media group (from 26.9 to 39.7), and among females (54%) in the Food Preparation and Serving Related group (SOC 35) (from 6.1 to 9.4). Rate changes among females in six SOC major groups were not reported because of small numbers (≤20 decedents in one or both years).

**TABLE 2:** Number and percentage of suicide decedents* in Standard Occupational Classification (SOC) major group, by year and sex — National Violent Death Reporting System, 17 states,^†^ 2012 and 2015

SOC code	Occupational group	Male		Female	
		2012 no. (%)	2015 no. (%)	2012 no. (%)	2015 no. (%)
11	Management	534 (8)	611 (9)	117 (7)	118 (7)
13	Business and Financial Operations	155 (2)	145 (2)	81 (5)	84 (5)
15	Computer and Mathematical	208 (3)	237 (3)	22 (1)	32 (2)
17	Architecture and Engineering	172 (3)	167 (2)	10 (1)	15 (1)
19	Life, Physical, and Social Science	56 (1)	52 (1)	15 (1)	21 (1)
21	Community and Social Service	41 (1)	48 (1)	39 (2)	40 (2)
23	Legal	54 (1)	49 (1)	34 (2)	29 (2)
25	Education, Training, and Library	91 (1)	87 (1)	82 (5)	84 (5)
27	Arts, Design, Entertainment, Sports, and Media	140 (2)	186 (3)	54 (3)	76 (4)
29	Health Care Practitioners and Technical occupations	145 (2)	169 (2)	220 (14)	225 (12)
31	Health Care Support	35 (1)	34 (<1)	97 (6)	124 (7)
33	Protective Service	232 (4)	226 (3)	29 (2)	32 (2)
35	Food Preparation and Serving Related	214 (3)	301 (4)	112 (7)	154 (9)
37	Building and Grounds Cleaning and Maintenance	316 (5)	315 (4)	36 (2)	46 (3)
39	Personal Care and Service	81 (1)	85 (1)	98 (6)	102 (6)
41	Sales and Related	555 (9)	553 (8)	170 (11)	212 (12)
43	Office and Administrative Support	244 (4)	260 (4)	234 (15)	268 (15)
45	Farming, Fishing, and Forestry	68 (1)	71 (1)	7 (<1)	5 (<1)
47	Construction and Extraction	1,216 (19)	1,404 (20)	12 (1)	17 (1)
49	Installation, Maintenance, and Repair	549 (9)	621 (9)	8 (1)	NR
51	Production	605 (9)	679 (10)	64 (4)	81 (4)
53	Transportation and Material Moving	736 (11)	817 (11)	52 (3)	39 (2)
NA	Military	228 (3)	203 (2)	15 (1)	13 (<1)
NA	Unpaid	822 (10)	913 (11)	724 (29)	795 (29)
NA	Insufficient Information to Classify Occupation	651 (8)	425 (5)	177 (9)	123 (4)

**Abbreviations:** NA = not assigned; NR = not reported due to cell size <5.

* Aged 16–64 years.

^†^ Alaska, Colorado, Georgia, Kentucky, Maryland, Massachusetts, New Jersey, New Mexico, North Carolina, Ohio, Oklahoma, Oregon, Rhode Island, South Carolina, Utah, Virginia, and Wisconsin.

**TABLE 3:** Suicide rate per 100,000 civilian, noninstitutionalized working persons aged 16–64 years, by sex, based on suicide decedents (N = 14,728) presumed in the labor force at time of death using Standard Occupational Classification (SOC) major groups — National Violent Death Reporting System, 17 states,* 2012 and 2015

Males						Females					
SOC code	Occupational group	2012	2015	Rate change		SOC code	Occupational group	2012	2015	Rate change	
				%	Rank^†^					%	Rank^†^
47	**Construction and Extraction**					27	**Arts, Design, Entertainment, Sports, and Media**				
	Rate rank^§^	1	1	+22%	5		Rate rank^§^	1	1	+34%	2
	Rate per 100,000	43.6	53.2				Rate per 100,000	11.7	15.6		
	95% CI^¶^	40.9–46.3	50.2–56.1				95% CI^¶^	8.6–15.5	12.1–19.8		
	Suicide decedents, no.	1,009	1,248				Suicide decedents, no.	47	67		
	Population, no.	2,313,934	2,345,952				Population, no.	403,305	429,424		
27	**Arts, Design, Entertainment, Sports, and Media**					33	**Protective Service**				
	Rate rank	7	2	+47%	1		Rate rank	2	2	+5%	9
	Rate per 100,000	26.9	39.7				Rate per 100,000	11.6	12.2		
	95% CI	22.1–31.8	33.6–45.8				95% CI	7.5–17.1	8.1–17.7		
	Suicide decedents, no.	117	162				Suicide decedents, no.	25	28		
	Population, no.	434,177	408,113				Population, no.	215,345	228,862		
49	**Installation, Maintenance, and Repair**					31	**Health Care Support**				
	Rate rank	2	3	+24%	3		Rate rank	5	3	+31%	3
	Rate per 100,000	31.6	39.1				Rate per 100,000	8.4	11.0		
	95% CI	28.7–34.4	35.8–42.3				95% CI	6.7–10.4	8.9–13.0		
	Suicide decedents, no.	473	542				Suicide decedents, no.	83	108		
	Population, no.	1,498,263	1,387,681				Population, no.	993,407	984,369		
53	**Transportation and Material Moving**					35	**Food Preparation and Serving Related**				
	Rate rank	4	4	+9%	8		Rate rank	11	4	+54%	1
	Rate per 100,000	28.4	30.9				Rate per 100,000	6.1	9.4		
	95% CI	26.2–30.7	28.6–33.1				95% CI	4.9–7.5	7.8–11.0		
	Suicide decedents, no.	615	721				Suicide decedents, no.	94	139		
	Population, no.	2,164,530	2,336,133				Population, no.	1,539,199	1,479,822		
51	**Production**					23	**Legal**				
	Rate rank	3	5	+7%	10		Rate rank	3	5	−17%	15
	Rate per 100,000	28.4	30.5				Rate per 100,000	11.1	9.2		
	95% CI	26.0–30.9	28.1–33.0				95% CI	7.5–15.9	5.8–13.9		
	Suicide decedents, no.	524	607				Suicide decedents, no.	30	22		
	Population, no.	1,843,879	1,987,864				Population, no.	269,243	238,870		
33	**Protective Service**					29	**Health Care Practitioners and Technical**				
	Rate rank	6	6	+4%	11		Rate rank	4	6	−13%	13
	Rate per 100,000	27.1	28.2				Rate per 100,000	10.3	9.0		
	95% CI	23.3–30.9	24.2–32.1				95% CI	8.9–11.8	7.7–10.3		
	Suicide decedents, no.	198	194				Suicide decedents, no.	195	193		
	Population, no.	730,044	689,034				Population, no.	1,890,885	2,140,217		
37	Building and Grounds Cleaning and Maintenance					51	**Production**				
	Rate rank	5	7	−2%	14		Rate rank	7	7	+18%	6
	Rate per 100,000	27.3	26.8				Rate per 100,000	7.6	9.0		
	95% CI	24.1–30.5	23.6–30.0				95% CI	5.8–10.0	7.0–11.3		
	Suicide decedents, no.	281	276				Suicide decedents, no.	55	72		
	Population, no.	1,028,779	1,029,385				Population, no.	719,183	800,640		
29	**Health Care Practitioners and Technical**					39	**Personal Care and Service**				
	Rate rank	14	8	+23%	4		Rate rank	9	8	+14%	7
	Rate per 100,000	20.8	25.6				Rate per 100,000	6.8	7.7		
	95% CI	17.1–24.6	21.5–29.8				95% CI	5.5–8.4	6.2–9.5		
	Suicide decedents, no.	119	145				Suicide decedents, no.	89	92		
	Population, no.	571,387	565,768				Population, no.	1,308,535	1,187,811		
45	**Farming, Fishing, and Forestry**					41	**Sales and Related**				
	Rate rank	8	9	−13%	21		Rate rank	10	9	+20%	5
	Rate per 100,000	26.3	22.8				Rate per 100,000	6.4	7.7		
	95% CI	20.0–34.0	17.7–29.0				95% CI	5.3–7.4	6.6–8.7		
	Suicide decedents, no.	58	67				Suicide decedents, no.	148	192		
	Population, no.	220,364	293,746				Population, no.	2,325,223	2,505,186		
41	**Sales and Related**					15	**Computer and Mathematical**				
	Rate rank	11	10	+1%	12		Rate rank	NR	10	NR	NR
	Rate per 100,000	21.3	21.5				Rate per 100,000	NR	7.3		
	95% CI	19.4–23.2	19.6–23.4				95% CI	NR	5.0–10.5		
	Suicide decedents, no.	487	489				Suicide decedents, no.	20	30		
	Population, no.	2,282,361	2,276,666				Population, no.	390,260	408,410		
35	**Food Preparation and Serving Related**					53	**Transportation and Material Moving**				
	Rate rank	19	11	+43%	2		Rate rank	6	11	−17%	14
	Rate per 100,000	14.6	20.9				Rate per 100,000	8.3	6.9		
	95% CI	12.5–16.7	18.4–23.3				95% CI	6.0–11.2	4.8–9.7		
	Suicide decedents, no.	180	276				Suicide decedents, no.	43	33		
	Population, no.	1,234,381	1,321,800				Population, no.	517,082	477,143		
31	**Health Care Support**					21	**Community and Social Service**				
	Rate rank	9	12	−12%	18		Rate rank	8	12	−17%	16
	Rate per 100,000	22.1	19.5				Rate per 100,000	7.3	6.0		
	95% CI	14.8–31.7	12.5–29.0				95% CI	5.1–10.2	4.2–8.4		
	Suicide decedents, no.	29	24				Suicide decedents, no.	34	36		
	Population, no.	131,497	123,003				Population, no.	464,942	595,582		
17	**Architecture and Engineering**					43	**Office and Administrative Support**				
	Rate rank	10	13	−10%	15		Rate rank	14	13	+27%	4
	Rate per 100,000	21.6	19.4				Rate per 100,000	4.7	6.0		
	95% CI	18.1–25.1	16.3–22.6				95% CI	4.1–5.4	5.2–6.8		
	Suicide decedents, no.	145	147				Suicide decedents, no.	201	239		
	Population, no.	670,938	756,515				Population, no.	4,267,892	3,985,105		
23	**Legal**					13	**Business and Financial Operations**				
	Rate rank	12	14	−12%	19		Rate rank	12	14	−5%	11
	Rate per 100,000	21.3	18.7				Rate per 100,000	5.7	5.4		
	95% CI	15.7–28.2	13.4–25.4				95% CI	4.4–7.2	4.2–6.8		
	Suicide decedents, no.	48	41				Suicide decedents, no.	70	71		
	Population, no.	225,681	219,171				Population, no.	1,235,880	1,321,724		
11	**Management**					37	**Building and Grounds Cleaning and Maintenance**				
	Rate rank	17	15	+8%	9		Rate rank	15	15	+14%	8
	Rate per 100,000	16.4	17.8				Rate per 100,000	4.6	5.2		
	95% CI	14.9–17.9	16.3–19.3				95% CI	3.1–6.5	3.7–7.2		
	Suicide decedents, no.	477	530				Suicide decedents, no.	31	36		
	Population, no.	2,906,468	2,981,498				Population, no.	673,483	688,809		
39	**Personal Care and Service**					11	**Management**				
	Rate rank	13	16	−21%	22		Rate rank	13	16	−12%	12
	Rate per 100,000	20.9	16.5				Rate per 100,000	5.6	4.9		
	95% CI	16.2–26.4	12.9–20.7				95% CI	4.5–6.7	4.0–5.9		
	Suicide decedents, no.	68	73				Suicide decedents, no.	104	103		
	Population, no.	326,037	443,543				Population, no.	1,855,055	2,083,968		
15	**Computer and Mathematical**					25	**Education, Training, and Library**				
	Rate rank	15	17	−11%	16		Rate rank	16	17	+3%	10
	Rate per 100,000	18.1	16.1				Rate per 100,000	3.3	3.4		
	95% CI	15.5–20.8	13.9–18.4				95% CI	2.6–4.2	2.7–4.2		
	Suicide decedents, no.	179	202				Suicide decedents, no.	69	74		
	Population, no.	986,994	1,252,275				Population, no.	2,091,706	2,186,483		
43	**Office and Administrative Support**					17	**Architecture and Engineering**				
	Rate rank	20	18	+12%	7		Rate rank	NR	NR	NR	NR
	Rate per 100,000	14.1	15.8				Rate per 100,000	NR	NR		
	95% CI	12.2–16.1	13.7–17.9				95% CI	NR	NR		
	Suicide decedents, no.	206	223				Suicide decedents, no.	10	12		
	Population, no.	1,456,242	1,411,453				Population, no.	135,632	144,852		
19	**Life, Physical, and Social Science**					19	**Life, Physical, and Social Science**				
	Rate rank	16	19	−13%	20		Rate rank	NR	NR	NR	NR
	Rate per 100,000	17.3	15.0				Rate per 100,000	NR	NR		
	95% CI	12.7–23.0	11.0–20.0				95% CI	NR	NR		
	Suicide decedents, no.	47	47				Suicide decedents, no.	13	19		
	Population, no.	271,690	312,925				Population, no.	225,992	204,566		
21	**Community and Social Service**					45	**Farming, Fishing, and Forestry**				
	Rate rank	21	20	+15%	6		Rate rank	NR	NR	NR	NR
	Rate per 100,000	12.8	14.6				Rate per 100,000	NR	NR		
	95% CI	8.8–17.9	10.7–19.6				95% CI	NR	NR		
	Suicide decedents, no.	33	45				Suicide decedents, no.	7	5		
	Population, no.	258,744	307,829				Population, no.	54,068	91,967		
13	**Business and Financial Operations**					47	**Construction and Extraction**				
	Rate rank	18	21	−11%	17		Rate rank	NR	NR	NR	NR
	Rate per 100,000	14.6	13.0				Rate per 100,000	NR	NR		
	95% CI	12.1–17.2	10.7–15.3				95% CI	NR	NR		
	Suicide decedents, no.	125	122				Suicide decedents, no.	9	14		
	Population, no.	855,329	941,806				Population, no.	55,164	76,173		
25	**Education, Training, and Library**					49	**Installation, Maintenance, and Repair**				
	Rate rank	22	22	−1%	13		Rate rank	NR	NR	NR	NR
	Rate per 100,000	10.9	10.9				Rate per 100,000	NR	NR		
	95% CI	8.6–13.6	8.6–13.5				95% CI	NR	NR		
	Suicide decedents, no.	78	79				Suicide decedents, no.	8	NR		
	Population, no.	713,321	727,167				Population, no.	73,231	46,136		

**Abbreviations**: CI = confidence interval; NR = not reported; number of decedents not reported <5, and rates were not calculated for occupational groups with ≤20 decedents; SOC = Standard Occupational Classification.

* Alaska, Colorado, Georgia, Kentucky, Maryland, Massachusetts, New Jersey, New Mexico, North Carolina, Ohio, Oklahoma, Oregon, Rhode Island, South Carolina, Utah, Virginia, and Wisconsin.

^†^ Rate change rank refers to each occupational group’s rank order for rate change from 2012 to 2015, where rank position 1 signifies the greatest suicide rate increase.

^§^ Occupational groups were ranked by 2015 suicide rate, separately for males and females. Because of rounding, some rate and rate change results are not precisely calculable from the data presented.

^¶^ 95% CIs were calculated using CDC’s National Center for Health Statistics methods, including confidence limit factors for mortality rates based on <100 decedents.

The 2012 and 2015 male suicide rates among Farmers, Ranchers, and Other Agricultural Managers (SOC 11–9013, a subgroup of the SOC 11 Management major group) were 44.9 (CI = 34.2–57.9) and 32.2 (CI = 24.2–42.0) per 100,000, based on 59 and 54 suicides in 2012 and 2015, respectively. The 2012 and 2015 male suicide rates for Agricultural Workers (SOC 45–2000, a subgroup of the SOC 45 Farming, Fishing, and Forestry major group) were 20.4 (CI = 13.8–29.1) and 17.3 (CI = 12.1–23.9), based on 30 and 36 suicides in 2012 and 2015, respectively.

## Discussion

Suicide rates varied widely across occupational groups in both 2012 and 2015, and rates among males and females increased in many occupational groups. The etiology of suicide is multifactorial, and identifying the specific role that occupational factors might play in suicide risk is complicated; both work (e.g., little job control or job insecurity) and nonwork (e.g., relationship conflict) factors are associated with psychological distress and suicide (*4*). The relationship between occupation and suicide might be confounded by access to lethal means on the job and socioeconomic factors such as lower income and education (*5*,*6*). Previous studies have employed a range of methodologies to study the proposed association between suicide and occupation and, at times, have arrived at different conclusions. For example, although this analysis aligns with another that found high suicide rates among construction workers in Colorado (*7*), a meta-analysis using an international occupational classification system found persons in other less-skilled occupations, such as laborers and cleaners, to be at higher risk (*6*).

A better understanding of how suicides are distributed by occupational group might help inform prevention programs and policies. Because many adults spend a substantial amount of their time at work, the workplace is an important but underutilized location for suicide prevention (*8*). Workplaces could potentially benefit from suicide prevention activities. Additional and tailored prevention approaches might be necessary to support workers at higher risk. Workplace suicide prevention efforts to date have focused primarily on early detection and tertiary intervention through the training of persons (i.e. gatekeepers) to identify those at risk for suicide and refer them to supporting services. However, more research on the role of the workplace in primary suicide prevention is needed, including improving working conditions and reducing stress (*8*).

The findings in this report are subject to at least four limitations. First, because of the nature of the data that were available, and consistent with previous research methods, this report compared decedents’ usual lifetime occupation as recorded on the death certificate with occupations of the employed population to calculate suicide rates. Additional data from coroner/medical examiner and law enforcement reports were used to exclude decedents identified as not in the labor force at time of death. Separate analyses indicated that if no such exclusion were applied, suicide rates would have been higher for all groups, although the top and bottom eight ranked occupational groups in 2015 by male suicide rate would maintain the same rank position, as would the top three and bottom four occupational groups by female suicide rate. Second, this report did not address confounding factors that might account for higher or lower rates of suicide between and within occupational groups, including education and income (*9*,*10*). Within SOC major occupational groups, employee education and income might vary widely. For example, the Management SOC major group includes farmers, ranchers, and chief executives of large companies, and the Construction and Extraction group includes both employees who might be salaried (e.g., supervisors) and those who might be paid hourly wages (e.g., roofer helpers). Future research might benefit from using more narrowly defined occupational groups and controlling for education and income to refine understanding of the relationship between occupation and suicide. Third, industry and occupation data obtained from death certificates rely on the accuracy and completeness of employment information provided by decedents’ family members. It is also possible that completeness and accuracy of that information might be associated with decedents’ job history. For example, categorization based on single lifetime industry and occupation might not accurately reflect employment for those persons with multiple lifetime occupations and those who worked across industries. Finally, this report is based on data from 17 U.S. states that participated in NVDRS in 2012 and 2015, and the data are not nationally representative.

To address the multifactorial etiology of suicide, CDC recommends a comprehensive approach to prevention (*1*). Strategies might include enhancing social connectedness and expanding access to relevant resources, strengthening state or local economic supports, implementing practices that encourage help-seeking and decrease stigma, and providing referrals to mental health and other services (*1*). Strategies can be implemented to assure support and reduce access to lethal means among persons at risk. Decision makers, including employers, can create a response plan, should a suicide affect their organization. Surviving family and friends can be supported to reduce their own suicide risk. The media can follow reporting recommendations to avoid sensationalized reporting and can refrain from providing details on suicide methods (*1*). Further workplace prevention resources are available at https://theactionalliance.org/, and help is available at 1-800-273-TALK.

**Summary**

**What is already known about this topic?**

From 2000 to 2016, the U.S. suicide rate among working aged (16–64 years) adults increased 34% from 12.9 per 100,000 population to 17.3.

**What is added by this report?**

2012 and 2015 National Violent Death Reporting System data from 17 states indicated the major occupational group with the highest male suicide rate was Construction and Extraction (43.6 [2012] and 53.2 [2015]). The Arts, Design, Entertainment, Sports, and Media major occupation group had the highest female suicide rate in 2012 (11.7) and 2015 (15.6).

**What are the implications for public health practice?**

A comprehensive approach to suicide prevention, including workplace-based approaches, is needed. CDC’s technical package of strategies to prevent suicide is a resource for communities and workplaces to identify prevention strategies with the best available evidence.

## Footnotes

* Alaska, Colorado, Georgia, Kentucky, Maryland, Massachusetts, New Jersey, New Mexico, North Carolina, Ohio, Oklahoma, Oregon, Rhode Island, South Carolina, Utah, Virginia, and Wisconsin.
