# *Erratum:* Vol. 67, No. 45

**DOI:** 10.15585/mmwr.mm6807a7

**Published:** 2019-02-22

In Table 2 of the report “Suicide Rates by Major Occupational Group — 17 States, 2012 and 2015,” on page 1255, some percentages were calculated using records with Standard Occupational Classification (SOC) codes as the denominator rather than all suicide decedents aged 16–64 years. The corrected Table 2 follows. The ranking of occupational groups by suicide rate is unaffected by this correction. On page 1254, the first sentence of the second complete paragraph should have read, “In both 2012 and 2015, the largest percentage of male suicides (**15%–16%** of decedents) occurred among those in the Construction and Extraction group (SOC 47) (Table 2); the largest percentage of female suicides in both years occurred among decedents with unpaid occupations (29%). The largest percentage of female suicides among classifiable occupations occurred in the Office and Administrative Support group (SOC 43) in both years (**9% and 10%**).”

**TABLE 2:** Number and percentage of suicide decedents* in Standard Occupational Classification (SOC) major group, by year and sex — National Violent Death Reporting System, 17 states,^†^ 2012 and 2015

SOC code	Occupational group	Male		Female	
		2012 no. (%)	2015 no. (%)	2012 no. (%)	2015 no. (%)
11	Management	534 (7)	611 (7)	117 (5)	118 (4)
13	Business and Financial Operations	155 (2)	145 (2)	81 (3)	84 (3)
15	Computer and Mathematical	208 (3)	237 (3)	22 (1)	32 (1)
17	Architecture and Engineering	172 (2)	167 (2)	10 (<1)	15 (1)
19	Life, Physical, and Social Science	56 (1)	52 (1)	15 (1)	21 (1)
21	Community and Social Service	41 (1)	48 (1)	39 (2)	40 (1)
23	Legal	54 (1)	49 (1)	34 (1)	29 (1)
25	Education, Training, and Library	91 (1)	87 (1)	82 (3)	84 (3)
27	Arts, Design, Entertainment, Sports, and Media	140 (2)	186 (2)	54 (2)	76 (3)
29	Health Care Practitioners and Technical occupations	145 (2)	169 (2)	220 (9)	225 (8)
31	Health Care Support	35 (<1)	34 (<1)	97 (4)	124 (5)
33	Protective Service	232 (3)	226 (3)	29 (1)	32 (1)
35	Food Preparation and Serving Related	214 (3)	301 (3)	112 (4)	154 (6)
37	Building and Grounds Cleaning and Maintenance	316 (4)	315 (4)	36 (1)	46 (2)
39	Personal Care and Service	81 (1)	85 (1)	98 (4)	102 (4)
41	Sales and Related	555 (7)	553 (6)	170 (7)	212 (8)
43	Office and Administrative Support	244 (3)	260 (3)	234 (9)	268 (10)
45	Farming, Fishing, and Forestry	68 (1)	71 (1)	7 (<1)	5 (<1)
47	Construction and Extraction	1,216 (15)	1,404 (16)	12 (<1)	17 (1)
49	Installation, Maintenance, and Repair	549 (7)	621 (7)	8 (<1)	NR (<1)
51	Production	605 (7)	679 (8)	64 (3)	81 (3)
53	Transportation and Material Moving	736 (9)	817 (9)	52 (2)	39 (1)
NA	Military	228 (3)	203 (2)	15 (1)	13 (<1)
NA	Unpaid	822 (10)	913 (11)	724 (29)	795 (29)
NA	Insufficient Information to Classify Occupation	651 (8)	425 (5)	177 (7)	123 (4)

**Abbreviations:** NA = not assigned; NR = not reported because cell size <5.

* Aged 16–64 years; column percentages do not sum to 100% because of rounding.

^†^ Alaska, Colorado, Georgia, Kentucky, Maryland, Massachusetts, New Jersey, New Mexico, North Carolina, Ohio, Oklahoma, Oregon, Rhode Island, South Carolina, Utah, Virginia, and Wisconsin.

